# Pharmacodynamic effects of cangrelor in patients with or without STEMI undergoing percutaneous coronary intervention: insights from the POMPEII study

**DOI:** 10.1093/ehjacc/zuag056

**Published:** 2026-04-15

**Authors:** Giuseppe Gargiulo, Viviana Narciso, Imma Forzano, Luca Sperandeo, Domenico Simone Castiello, Fiorenzo Simonetti, Lina Manzi, Domenico Florimonte, Mario Enrico Canonico, Marisa Avvedimento, Roberta Paolillo, Alessandra Spinelli, Luigi Di Serafino, Carmen Anna Maria Spaccarotella, Anna Franzone, Raffaele Piccolo, Eugenio Stabile, Plinio Cirillo, Marco Valgimigli, Giovanni Esposito

**Affiliations:** Division of Cardiology, Department of Advanced Biomedical Sciences, Federico II University of Naples, Via S. Pansini 5, Naples 80131, Italy; Division of Cardiology, Department of Advanced Biomedical Sciences, Federico II University of Naples, Via S. Pansini 5, Naples 80131, Italy; Division of Cardiology, Department of Advanced Biomedical Sciences, Federico II University of Naples, Via S. Pansini 5, Naples 80131, Italy; Division of Cardiology, Department of Advanced Biomedical Sciences, Federico II University of Naples, Via S. Pansini 5, Naples 80131, Italy; Division of Cardiology, Department of Advanced Biomedical Sciences, Federico II University of Naples, Via S. Pansini 5, Naples 80131, Italy; Division of Cardiology, Department of Advanced Biomedical Sciences, Federico II University of Naples, Via S. Pansini 5, Naples 80131, Italy; Division of Cardiology, Department of Advanced Biomedical Sciences, Federico II University of Naples, Via S. Pansini 5, Naples 80131, Italy; Division of Cardiology, Department of Advanced Biomedical Sciences, Federico II University of Naples, Via S. Pansini 5, Naples 80131, Italy; Division of Cardiology, Department of Advanced Biomedical Sciences, Federico II University of Naples, Via S. Pansini 5, Naples 80131, Italy; CPC Clinical Research, University of Colorado, Aurora, CO, USA; Division of Cardiology, Department of Advanced Biomedical Sciences, Federico II University of Naples, Via S. Pansini 5, Naples 80131, Italy; Division of Cardiology, Department of Advanced Biomedical Sciences, Federico II University of Naples, Via S. Pansini 5, Naples 80131, Italy; Division of Cardiology, Department of Advanced Biomedical Sciences, Federico II University of Naples, Via S. Pansini 5, Naples 80131, Italy; Division of Cardiology, Department of Advanced Biomedical Sciences, Federico II University of Naples, Via S. Pansini 5, Naples 80131, Italy; Division of Cardiology, Department of Advanced Biomedical Sciences, Federico II University of Naples, Via S. Pansini 5, Naples 80131, Italy; Division of Cardiology, Department of Advanced Biomedical Sciences, Federico II University of Naples, Via S. Pansini 5, Naples 80131, Italy; Division of Cardiology, Department of Advanced Biomedical Sciences, Federico II University of Naples, Via S. Pansini 5, Naples 80131, Italy; Cardiovascular Department, Azienda Ospedaliera Regionale ‘San Carlo,’ Potenza, Italy; Division of Cardiology, Department of Advanced Biomedical Sciences, Federico II University of Naples, Via S. Pansini 5, Naples 80131, Italy; Cardiocentro Institute, Ente Ospedaliero Cantonale, Università Della Svizzera Italiana (USI), Lugano, Switzerland; Division of Cardiology, Department of Advanced Biomedical Sciences, Federico II University of Naples, Via S. Pansini 5, Naples 80131, Italy

**Keywords:** Cangrelor, Percutaneous coronary intervention, STEMI, Acute coronary syndrome, Pharmacodynamic, Platelet inhibition

## Abstract

**Aims:**

Cangrelor is approved for oral P2Y12 inhibitor naïve patients undergoing percutaneous coronary intervention (PCI). Pharmacodynamic (PD) investigations in different clinical settings, using various assays, have shown contrasting data in terms of entity of platelet inhibition and rates of high residual platelet reactivity (HRPR). We assessed the PD effects in patients with or without ST elevation myocardial infarction (STEMI) receiving cangrelor during PCI.

**Methods and results:**

The PharmacOdynaMic effects of cangrelor in PatiEnts wIth acute or chronIc coronary syndrome undergoing percutaneous coronary intervention (POMPEII) registry (NCT04790032) is an investigator-initiated, prospective study assessing PD at four time points with three assays. From March 2021 to June 2024, 126 patients naïve from oral P2Y12 inhibitors underwent PCI with cangrelor [32 with STEMI and 94 without (NSTE-ACS = 30, CCS = 64)]. All STEMI patients switched from cangrelor to ticagrelor, while most patients without STEMI switched to clopidogrel. Inhibition of platelet aggregation (IPA%) during cangrelor infusion was lower in patients with STEMI compared with those without STEMI (LTA 20-μM-ADP 51.5 ± 16.2% vs. 59.7 ± 16.2%; *P* = 0.017). Conversely, after switching from cangrelor to an oral P2Y12 inhibitor, IPA was greater in patients with STEMI compared with those without STEMI. Rates of HRPR were consistent with lower platelet inhibition in STEMI during cangrelor and greater after its discontinuation compared with patients without STEMI. Within STEMI patients, cangrelor-induced IPA was lower compared with ticagrelor-induced IPA (*P* = 0.036).

**Conclusion:**

Cangrelor-induced platelet inhibition was lower in patients with STEMI compared with those without STEMI and was lower than that induced by ticagrelor among STEMI patients. The switch from cangrelor to an oral P2Y12 inhibitor exposed patients without STEMI to greater HRPR compared with those with STEMI.

## Graphical Abstract

**Graphical Abstract:**
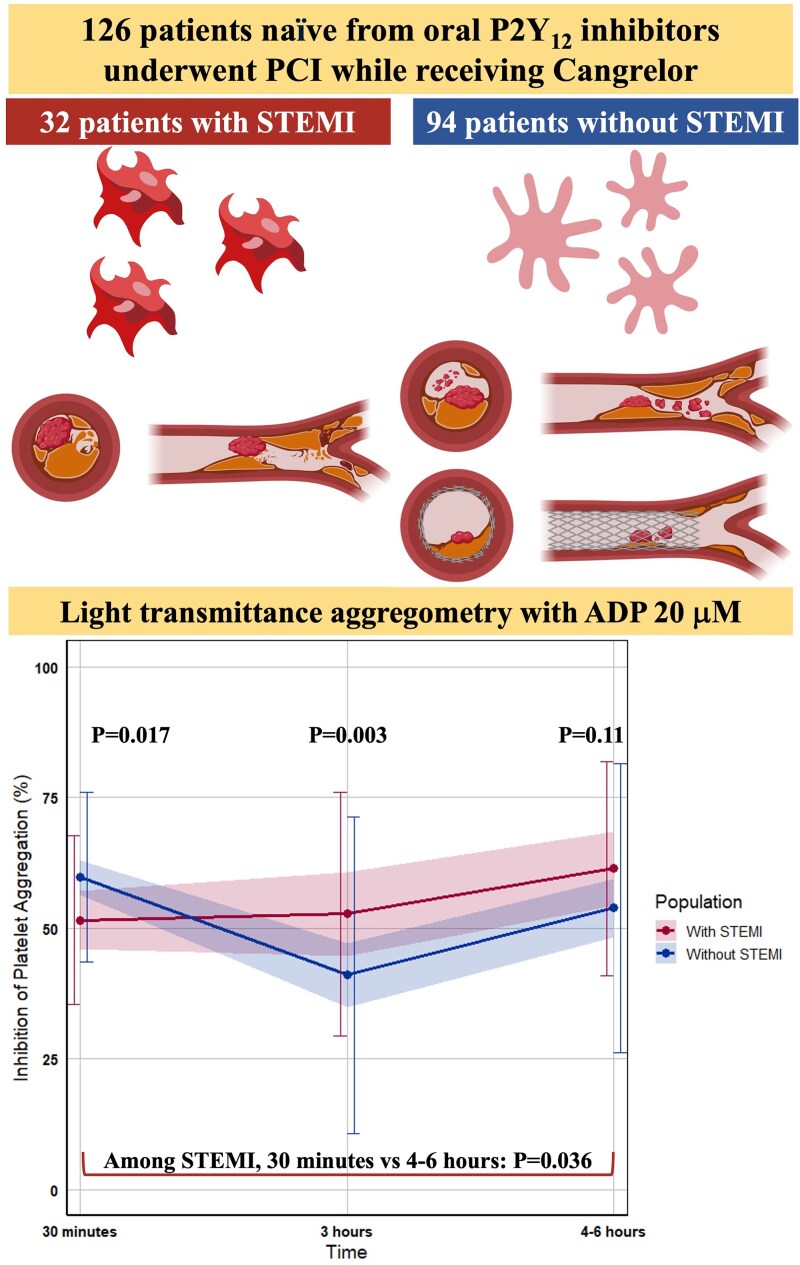
Main findings of the POMPEII study in patients with or without STEMI. Inhibition of platelet aggregation at 30 min, 3 h, and 4–6 h with light transmittance aggregometry (LTA 20-μM ADP) is shown.

## Introduction

Cangrelor is approved for patients with acute coronary syndrome (ACS) or chronic coronary syndrome (CCS) who are naïve from oral P2Y12 inhibitors and are undergoing percutaneous coronary intervention (PCI).^1–3^ Its intravenous administration with rapid onset and offset of platelet inhibition at the time of PCI is particularly intriguing in the setting of ACS. Indeed, oral agents require some hours to achieve their antiplatelet effect, and their use as pretreatment has been even downgraded or not recommended in most patients with ST-segment elevation myocardial infarction (STEMI) or NSTE-ACS, respectively.^1^ In the setting of CCS patients undergoing elective complex PCI, an adequate platelet inhibition in the periprocedural phase is also warranted.^2,4^ However, thrombotic risks are different between CCS and ACS, and within ACS, distinct clinical entities present with varying thrombotic risk profiles. Specifically, patients presenting with STEMI exhibit unique demographic and pathophysiological features and are characterized by a large thrombotic burden at presentation.^5,6^

Several studies have explored pharmacodynamic (PD) effects of cangrelor across different clinical settings, using different platelet reactivity assays and with some contrasting evidence in terms of magnitude of platelet inhibition and rates of high residual platelet reactivity (HRPR), particularly in STEMI patients.^7–10^ Moreover, the switching from cangrelor to an oral P2Y12 inhibitor (clopidogrel, ticagrelor, prasugrel) has also generated concerns regarding the type and timing of the selected oral agent due to potential drug–drug interactions.^4,11–13^ Hence, contemporary PD data are needed. In the recent prospective PharmacOdynaMic effects of cangrelor in PatiEnts wIth acute or chronIc coronary syndrome undergoing percutaneous coronary intervention (POMPEII) registry, we have shown the PD effects of cangrelor in all patients.^14^ Here, we analysed the POMPEII prospective registry of all patients undergoing PCI with cangrelor to carefully investigate the PD effects of cangrelor in patients with or without STEMI.

## Methods

### Study design and patient population

The PharmacOdynaMic effects of cangrelor in PatiEnts wIth acute or chronIc coronary syndrome undergoing percutaneous coronary intervention (POMPEII) registry (NCT04790032) is an investigator-initiated, prospective, single-centre study conducted at Federico II University of Naples.^4,14,15^ All adult patients undergoing PCI and receiving cangrelor at operator’s discretion were eligible. Patients were included if they provided consent to blood/data collection and if the study team was available for analyses. No specific exclusion criteria were applied.

All demographic, clinical, procedural, and therapeutic data of patients were collected. The RedCap electronic data capture system was used. The study complied with the Declaration of Helsinki and was approved by the internal Ethics Committee. All patients provided written informed consent.

### Medications and procedures

All patients received aspirin, unfractionated heparin, and cangrelor (30 μg/kg bolus followed by 4 μg/kg/min infusion for 2 h) prior to the start of PCI that was performed according to most recent guidelines and standard of care. Eventually needed as bailout, the glycoprotein IIb/IIIa inhibitor (GPI) tirofiban was administered at a 25 μg/kg bolus with or without 0.15 μg/kg per minute (or 0.075 μg/kg per minute if creatinine clearance was <30 mL/min) infusion (the use of infusion and its duration were at operator’s discretion), and PD analyses were interrupted. Ticagrelor, prasugrel, and clopidogrel were administered as loading dose and maintenance dose according to guidelines. The type of oral P2Y12 inhibitor and the timing of its loading dose in the transition from cangrelor were also left at operator’s discretion.

### Pharmacodynamic assessment

Pharmacodynamic assessments were performed with three assays as previously described^14^: 1) the gold standard light transmittance aggregometry (LTA) (20- and 5-μM ADP stimuli), 2) VerifyNow P2Y12-test, and 3) multiplate electrode aggregometry (MEA), ADP test.

Blood samples for PD assessments were collected at baseline (before cangrelor bolus administration), 30 min, 3 h (thus, 1 h after cangrelor infusion stop), and 4–6 h (thus, 2–4 h after cangrelor infusion stop) after cangrelor initiation. All PD tests were performed within 30 min from blood collection by experienced laboratory personnel. High residual platelet reactivity standard definitions were used.

- LTA: Light transmittance aggregometry was performed as previously described. Briefly, ADP (5 and 20 μM) was used as a pro-aggregatory stimulus. The results as a percentage of maximum platelet aggregation (MPA) were collected and used to calculate the percentage of inhibition of platelet aggregation (IPA%). High residual platelet reactivity was defined as MPA >59% (using LTA 20 μmol/L ADP) and MPA >46% (using LTA 5 μmol/L).
- MEA: Multiplate electrode aggregometry was assessed in the whole blood by the Multiplate analyzer (Roche-Dynabyte Medical, Munich, Germany). Briefly, ADPtest was used to assess ADP-induced pathway. The mean values of two independent determinations were expressed as area under curve (AUC) in arbitrary units (U; 1 U = 10 AU min, aggregation units minutes), maximal aggregation (AU), and velocity (AU/min). High residual platelet reactivity was defined as AUC >46 U (MEA-ADP).
- VerifyNow: The VerifyNow-P2Y12 assay (Accumetrics, San Diego, California) measures ADP-induced platelet agglutination as an increase in light transmittance and utilizes a proprietary algorithm to report values in P2Y12 reaction units (PRU). High residual platelet reactivity was defined as PRU >208.

### Study PD and clinical outcomes

The primary outcome was the 30-minute percentage of IPA (%IPA) assessed with LTA after stimulation of platelet-rich plasma with ADP 20 μmol/L as previously described.^10^ %IPA is defined as follows: 100% × (baseline platelet aggregation − platelet aggregation at time t)/baseline platelet aggregation. Secondary outcomes included all the values of MPA and %IPA measured at various time points with LTA with ADP 20 μmol/L and with ADP 5 μmol/L, as well as PRU values measured with P2Y12 test at VerifyNow and AUC values (residual platelet reactivity) measured by Multiplate after stimulation with ADP at all time points.

Data on clinical outcomes (death, cardiovascular death, myocardial infarction, stent thrombosis, stroke, transient ischaemic attack, urgent revascularization, bleeding) during the periprocedural phase and up to 30 days by phone calls or in person visits were collected. Standard definitions were used for these clinical events that were blindly adjudicated by an independent clinical event committee composed of two cardiologists not involved in patients’ recruitment or management, as previously described.^14^

### Statistical analysis

No specific sample size was prespecified. Most PD studies on P2Y12 inhibitors, including cangrelor, enrolled a variable number between 15 and 50 patients or group of patients to explore differences in platelet inhibition. Aiming to include all various clinical settings (CCS and ACS subtypes), and different combinations of oral P2Y12 inhibitors, we planned to prospectively enrol at least 100 patients to allow the exploration of PD effects across these settings.

Data were presented as proportions, medians with interquartile range (IQR), or mean ± SD as appropriate. Differences in categorical variables between respective comparison groups were analysed using the chi-square statistic test (χ2) or Fisher exact test as appropriate. The continuous variables were compared using Student’s *t*-test and Mann–Whitney U test. To evaluate the temporal evolution of platelet reactivity across groups, we also used a linear mixed-effects model: time, clinical presentation (STEMI vs. without STEMI), and their interaction (time × presentation) were used as fixed effects; patient identifier as a random intercept; the 30 min time point as the reference level, and without-STEMI was the reference category for clinical presentation. IPA was calculated as stated above. This analysis included 126 patients excluding 24 of the total of 150 patients because they were STEMI who received ticagrelor pretreatment, thus affecting IPA interpretation. Indeed, IPA is calculated using the baseline platelet aggregation that in pretreated patients is affected by ticagrelor pretreatment. A two-tailed α-value of <0.05 was considered significant. Statistical analyses were performed using SPSS (version 29.0; IBM, Chicago, IL, USA) and R statistical software (version 4.1.2).

## Results

From March 2021 to June 2024, 150 patients undergoing PCI and receiving cangrelor were enrolled in the study of whom 126 were naïve from oral P2Y12 inhibitors and included in the present analysis. Among them, 32 (25.4%) presented with STEMI and 94 (74.6%) without STEMI (CCS = 64, 68.1%; NSTE-ACS = 30, 31.9%).

Mean age of the study population was 67.8 ± 10.5 years, and 23.8% were female. Compared with those without STEMI, patients with STEMI less frequently had history of hypertension and hyperlipidaemia or were on chronic therapy with aspirin, statins, and proton pump inhibitors. ST elevation myocardial infarction patients presented more frequently with lower LVEF, greater heart rate, and Killip class and had lower treated lesions, number of stents implanted, and total length of stent (*Tables 1* and *2*). Overall, two patients required bailout with tirofiban due to intraprocedural thrombotic complications, one among the STEMI (3.1%) and one among those without (1.1%). Clinical and procedural characteristics in patients with or without STEMI are reported in *Tables 1* and *2*.

**Table 1:** Baseline characteristics of the study population

Characteristics	Overall population (*n* = 126)	Patients with STEMI (*n* = 32)	Patients without STEMI (*n* = 94)	*P* value
Age, y	67.8 ± 10.5	66.2 ± 14.6	68.4 ± 8.7	0.43
Female	30 (23.8%)	10 (31.3%)	20 (21.3%)	0.25
Body mass index, Kg/m^2^	27.5 ± 4.1	27.5 ± 4.8	27.5 ± 3.9	0.94
Current smoking	44 (34.9%)	14 (43.8%)	30 (31.9%)	0.23
Hypertension	105 (83.3%)	21 (65.6%)	84 (89.4%)	0.002
Diabetes mellitus	42 (33.0%)	7 (21.9%)	35 (37.2%)	0.11
Hyperlipidaemia	82 (65.1%)	11 (34.4%)	71 (75.5%)	0.001
Family history of premature CAD	24 (19.0%)	7 (21.9%)	17 (18.1%)	0.64
Peripheral arterial disease	9 (7.1%)	1 (3.1%)	8 (8.5%)	0.31
Carotid artery disease	15 (11.9%)	5 (15.6%)	10 (10.6%)	0.45
Prior MI	21 (16.7%)	4 (12.5%)	17 (18.1%)	0.46
Prior PCI	27 (21.4%)	5 (15.6%)	22 (23.4%)	0.35
Prior CABG	7 (5.6%)	3 (9.4%)	4 (4.3%)	0.28
Prior stroke	5 (4.0%)	1 (3.1%)	4 (4.3%)	0.78
Prior TIA	4 (3.2%)	2 (6.3%)	2 (2.1%)	0.25
Previous bleeding requiring medical attention	3 (2.4%)	1 (3.1%)	2 (2.1%)	0.75
Congestive heart failure	15 (11.9%)	1 (3.1%)	14 (14.9%)	0.08
Left ventricular ejection fraction	49.1 ± 9.5	43.7 ± 8.3	51.0 ± 9.2	0.001
Chronic kidney disease (eGFR <60 mL/min)	19 (15.1%)	8 (25.0%)	11 (11.7%)	0.07
Chronic obstructive pulmonary disease	19 (15.1%)	3 (9.4%)	16 (17.0%)	0.30
Baseline Medications				
Aspirin (daily dose ≤100 mg)	75 (59.5%)	11 (34.4%)	64 (68.1%)	0.001
Oral anticoagulants	9 (7.1%)	1 (3.1%)	8 (8.5%)	0.40
Statins	70 (55.6%)	9 (28.1%)	61 (64.9%)	0.001
Other lipid-lowering drug	23 (19.8%)	4 (12.5%)	21 (22.3%)	0.55
ACE inhibitor	35 (27.8%)	8 (25.0%)	27 (28.7%)	0.69
ATII antagonist	49 (38.9%)	8 (25.0%)	41 (43.6%)	0.06
Beta blocker	56 (44.4%)	10 (31.3%)	46 (48.9%)	0.08
Amiodarone	6 (4.8%)	1 (3.1%)	5 (5.3%)	0.62
Ca-antagonist	39 (31.0%)	8 (25.0%)	31 (33.0%)	0.40
Nitrates	3 (2.4%)	1 (3.1%)	2 (2.1%)	0.75
Diuretics	40 (31.0%)	8 (25.0%)	32 (34.0%)	0.34
Insulin	13 (10.3%)	1 (3.1%)	12 (12.8%)	0.12
Oral antidiabetic	35 (27.8)	5 (15.6%)	30 (31.9%)	0.08
NSAID	3 (2.4%)	1 (3.1%)	2 (2.1%)	0.75
Antidepressant drug	5 (4.0%)	1 (3.1%)	4 (4.3%)	0.78
Proton pump inhibitors	74 (58.7%)	10 (31.3%)	64 (68.1%)	0.001
Haemoglobin, g/dL	13.6 ± 1.8	13.7 ± 1.9	13.6 ± 1.8	0.88
Creatinine, mg/dL	1.0 ± 0.5	1.2 ± 0.7	1.0 ± 0.5	0.52
Platelet count, ×1000/mm^3^	228.8 ± 84.9	252.0 ± 96.9	225.5 ± 83.1	0.36

Data are numbers (%) or mean (standard deviation).

ACE, angiotensin II converting enzyme; CABG, coronary artery bypass graft; CAD, coronary artery disease; eGFR, estimated glomerular filtration rate; MI, myocardial infarction; NSAID, nonsteroidal anti-inflammatory drug; PCI, percutaneous coronary intervention; TIA, transient ischaemic attack.

**Table 2:** Clinical presentation and procedural characteristics of the study population

Characteristics	Overall population (*n* = 126)	Patients with STEMI (*n* = 32)	Patients without STEMI (*n* = 94)	*P* value
Clinical presentation				
STEMI	32 (25.4%)	32 (100%)	—	
NSTE-ACS	30 (23.8%)	—	30 (31.9%)	
Elective PCI	64 (50.8%)	—	64 (68.1%)	
Systolic arterial pressure—mmHg	135.2 ± 18.6	129.6 ± 21.9	137.1 ± 17.1	0.09
Diastolic arterial pressure—mmHg	77.7 ± 13.2	78.1 ± 16.8	77.6 ± 11.8	0.88
Heart rate—beats/min	71.7 ± 14.5	80.1 ± 14.6	68.9 ± 13.4	0.001
Killip class				0.004
I	101 (80.2%)	20 (62.5%)	81 (86.2%)	
II	20 (15.9%)	8 (25.0%)	12 (12.8%)	
III	2 (1.6%)	1 (3.1%)	1 (1.1%)	
IV	3 (2.4%)	3 (9.4%)	0	
Rhythm at presentation				
Sinus rhythm	123 (97.6%)	32 (100%)	91 (96.8%)	0.31
Atrial fibrillation	2 (1.6%)	0	2 (2.1%)	0.41
Other rhythm	1 (0.8%)	0	1 (1.1%)	0.56
Intraventricular conduction defects				
Left-bundle branch block	7 (5.6%)	1 (3.1%)	6 (6.4%)	0.49
Right-bundle branch block	8 (6.3%)	3 (9.4%)	5 (5.3%)	0.42
**Catheterization**				
Radial artery access	111 (88.1%)	26 (81.3%)	85 (90.4%)	0.17
Hemodynamic support	2 (1.6%)	0	2 (2.1%)	0.56
Multivessel disease	79 (62.7%)	17 (53.1%)	62 (66.0%)	0.20
Vessel treated:				0.07
LM	7 (5.5%)	1 (3.1%)	6 (6.4%)	
LAD	68 (54.0%)	16 (50.0%)	52 (55.3%)	
LCX	22 (17.5%)	7 (21.9%)	15 (16.0%)	
RCA	29 (23.0%)	8 (25.0%)	21 (22.3%)	
PCI success	124 (98.4%)	32 (100%)	92 (97.8%)	0.41
Total number of lesions treated	1.4 ± 0.7	1.2 ± 0.5	1.5 ± 0.8	0.01
Total number of stents implanted	1.7 ± 1.0	1.3 ± 0.6	1.8 ± 1.1	0.006
Total length of stents implanted, mm	41.4 ± 24.6	33.9 ± 17.7	44.0 ± 26.2	0.02

Data are numbers (%) or mean (standard deviation).

LAD, left anterior descending; LCX, left circumflex; LIMA, left internal mammary artery; LM, left main coronary artery; NSTE-ACS, non-ST elevation acute coronary syndrome; PCI, percutaneous coronary intervention; RCA, right coronary artery; STEMI, ST elevation myocardial infarction.

All patients were switched to an oral P2Y12 inhibitor after initiation of cangrelor infusion, except for one, who received prolonged cangrelor infusion and was transferred for CABG. All STEMI patients switched to ticagrelor (100%). Among patients without STEMI, 29 (31.2%) switched to ticagrelor (23 NSTEMI and 6 elective PCI), 61 (65.6%) received clopidogrel (5 NSTEMI and 56 elective PCI) at the end of cangrelor infusion (loading dose of 600 mg in 59 patients and 300 mg in 2 patients), and 3 (3.2%) received prasugrel (2 NSTEMI and 1 elective PCI) at the end of the cangrelor infusion. After the initiation of cangrelor infusion, ticagrelor was administered at a mean of 51.0 ± 42.6 min in patients with STEMI and 51.8 ± 30.8 min in those without STEMI (*P* = 0.93).

### Pharmacodynamic assessment

Pharmacodynamic samples were analysed in all patients at every time point. Samples at 3 h and 4–6 h were not collected only in the two patients in whom bailout tirofiban was needed.

#### Pharmacodynamic assessment at baseline

Baseline values were not different and almost overlapping among patients with or without STEMI: LTA 20-μM-ADP MPA 79.1%±10.9% vs. 76.9%±11.3%, *P* = 0.36; LTA 5-μM-ADP MPA 65.9%±12.2% vs. 64.4%±13.6%, *P* = 0.57; MEA-AUC 64.3 ± 18.8 vs. 59.8 ± 15.6, *P* = 0.19; PRU 208.1 ± 50.1 vs. 216.5 ± 49.4, *P* = 0.41.

#### Pharmacodynamic assessment at 30 min (during cangrelor infusion)

**Figure 1:**
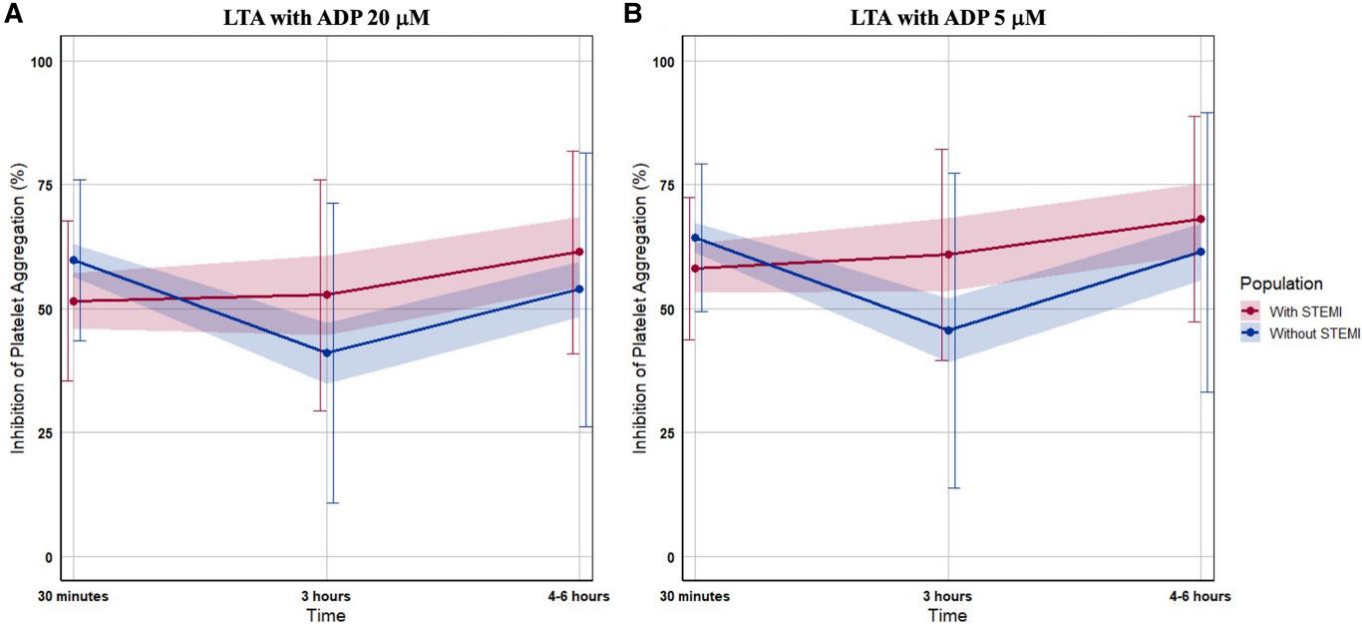
**Inhibition of platelet aggregation at light transmittance aggregometry.** Platelet inhibition assessment at 30 min, 3 h, and 4–6 h by light transmittance aggregometry (Panel A with LTA 20-μM and Panel B with ADP 5-μM) in patients with (red line) and without STEMI (blue line). ADP, adenosine diphosphate; LTA, light transmittance aggregometry.

**Figure 2:**
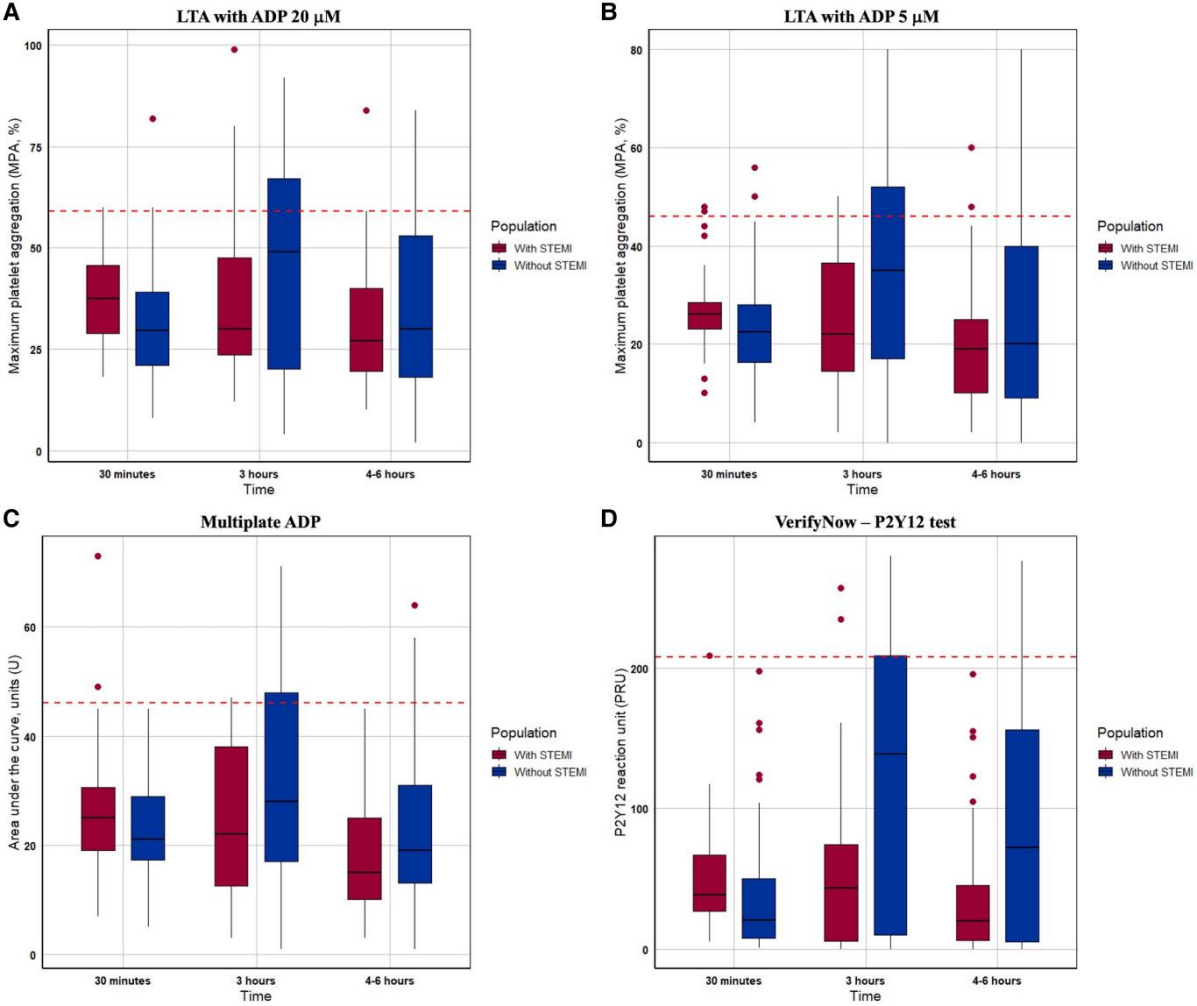
**Pharmacodynamic data with all tests at different time points.** Box plots of values of platelet aggregation at 30 min, 3 h, and 4–6 h as assessed with LTA (Panel A with ADP 20 and Panel B with ADP 5 μM), Multiplate (*C*), and VerifyNow (*D*). ADP, adenosine diphosphate; LTA, light transmittance aggregometry.

**Figure 3:**
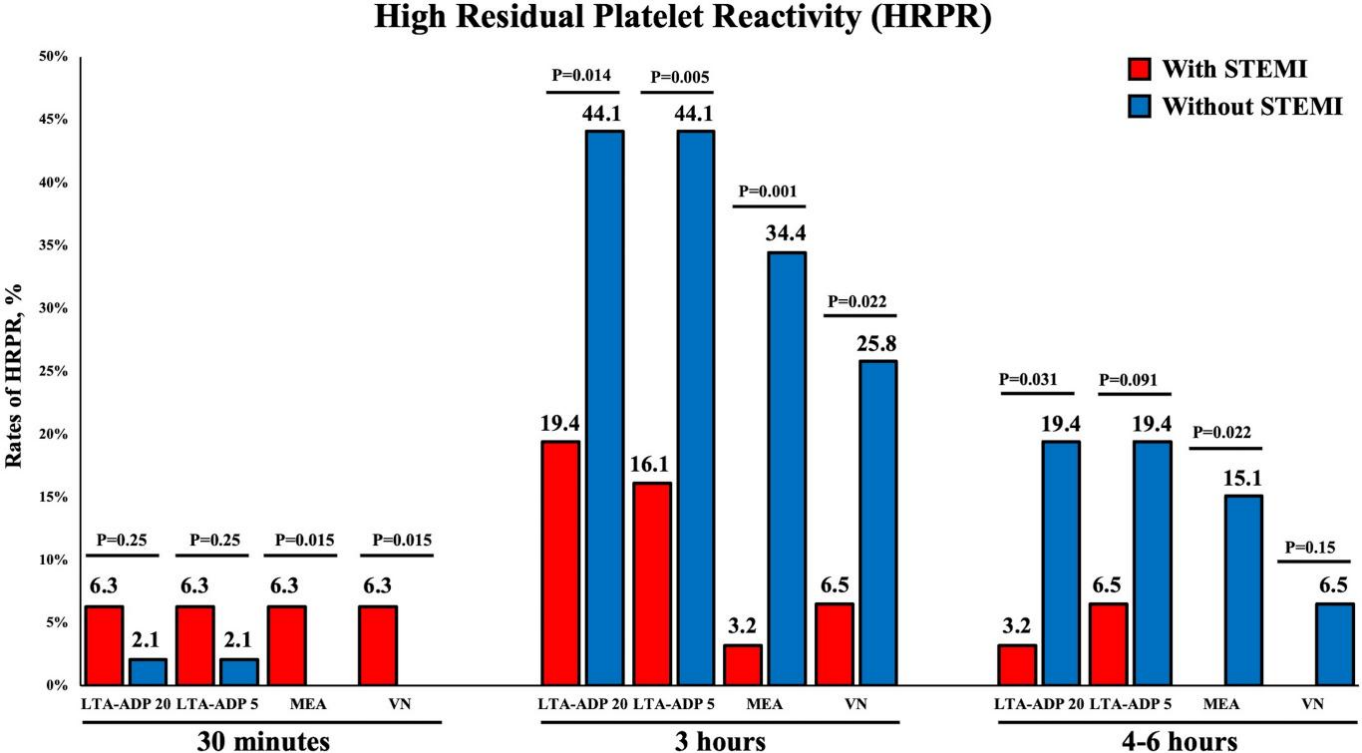
**Rates of HRPR.** Rates of HRPR at 30 min, 3 h, and 4–6 h with all tests defined as 20-μM ADP-induced maximal platelet aggregation >59%, 5-μM ADP-induced maximal aggregation >46%, AUC >46 U, and PRU > 208, respectively. ADP, adenosine diphosphate; HRPR, high residual platelet reactivity; LTA, light transmittance aggregometry; MEA, multiplate electrode aggregometry (area under the curve); STEMI, ST-elevation myocardial infarction; VN, VerifyNow (P2Y12 reaction unit).

At 30 min during cangrelor infusion, the mean IPA at LTA 20-μM-ADP was 51.5 ± 16.2% (0% with IPA >80%) vs. 59.7 ± 16.2% (6.4% with IPA >80%) in patients with and without STEMI, respectively (*P* = 0.017) (*Figure 1A*). At LTA 5-μM-ADP was 58.1 ± 14.3% (9.4% with IPA >80%) vs. 64.3 ± 14.9% (10.6% with IPA >80%) in patients with and without STEMI, respectively (*P* = 0.043) (*Figure 1B*). MEA-AUC was 26.7 ± 12.7 vs. 22.9 ± 9.3, respectively (*P* = 0.13) (*Figure 2*). P2Y12 reaction unit was 55.4 ± 51.5 vs. 35.6 ± 38.9, respectively (*P* = 0.024) (*Figure 2*). There were two cases (6.3%) vs. two cases (2.1%) of HRPR among patients with and without STEMI at LTA with both stimuli (*P* = 0.25), while there were two (6.3%) vs. zero HRPR (*P* = 0.015) with both MEA and VerifyNow (*Figure 3*).

#### Pharmacodynamic assessment at 3 h (1 h after cangrelor discontinuation)

At 3 h, the mean IPA was 52.7 ± 23.3% vs. 41.0 ± 30.2% (*P* = 0.029) at LTA 20-μM-ADP and 60.9 ± 21.3% vs. 45.5 ± 31.8% (*P* = 0.003) at LTA 5-μM-ADP in patients with and without STEMI, respectively (*Figure 1*). MEA-AUC was 24.5 ± 12.7 vs. 31.8 ± 16.7 (*P* = 0.012) and PRU was 56.5 ± 68.3 vs. 120.7 ± 90.8 (*P* < 0.0001), respectively (*Figure 2*). High residual platelet reactivity was observed in six cases (19.4%) vs. 41 (44.1%) in patients with and without STEMI, respectively (*P* = 0.014), at LTA 20-μM-ADP; in five cases (16.1%) vs. 41 (44.1%), respectively (*P* = 0.005) at LTA 5-μM-ADP; in one case (3.2%) vs. 32 (34.4%), respectively (*P* = 0.001) at MEA; and in two cases (6.5%) vs. 24 (25.8%), respectively (*P* = 0.02) at VerifyNow (*Figure 3*).

#### Pharmacodynamic assessment at 4–6 h (2–4 h after cangrelor discontinuation)

At 4–6 h, the mean IPA was 61.4 ± 20.5% vs. 53.8 ± 27.7% (*P* = 0.11) at LTA 20-μM-ADP and 68.0 ± 20.7% vs. 61.4 ± 28.2% (*P* = 0.16) at LTA 5-μM-ADP in patients with and without STEMI, respectively (*Figure 1*). MEA-AUC was 18.4 ± 10.8 vs. 23.6 ± 15.3 (*P* = 0.042) and PRU was 42.6 ± 52.6 vs. 88.7 ± 79.6 (*P* < 0.0001), respectively (*Figure 2*). High residual platelet reactivity was observed in 1 case (3.2%) vs. 18 (19.4%) in patients with and without STEMI, respectively (*P* = 0.03), at LTA 20-μM-ADP; in 2 cases (6.5%) vs. 18 (19.4%), respectively (*P* = 0.09) at LTA 5-μM-ADP; in 0 cases (0%) vs. 14 (15.1%), respectively (*P* = 0.02) at MEA; and in 0 cases (0%) vs. 6 (6.5%), respectively (*P* = 0.14) at VerifyNow (*Figure 3*).

#### Pharmacodynamic assessment at 30 min vs. 4–6 h in STEMI

Among patients with STEMI, the platelet inhibition at 30 min during cangrelor infusion was lower compared with ticagrelor-induced platelet inhibition at 4–6 h: IPA at LTA 20-μM-ADP *P* = 0.036; 5-μM-ADP *P* = 0.029; MEA-AUC *P* = 0.007, PRU *P* = 0.3 (*Figures 1* and *2*).

#### Additional pharmacodynamic assessments

Results were consistent when additional analyses were performed (see Supplementary material online, *Appendix*).

### Clinical outcomes

Clinical outcomes at 30 days were similar among groups (*Table 3*).

**Table 3:** Clinical outcomes at 30 days

	Overall population (*n* = 126)	Patients with STEMI (*n* = 32)	Patients without STEMI (*n* = 94)
All-cause death	3 (2.4%)	2 (6.3%)	1 (1.1%)
Cardiovascular death	1 (0.8%)	0	1 (1.1%)
Myocardial infarction	1 (0.8%)	0	1 (1.1%)
Stroke or transient ischaemic attack	0	0	0
Stent thrombosis	0	0	0
Bleeding events	15 (11.9%)	3 (9.4%)	12 (12.8%)
Requiring transfusion	2 (1.6%)	1 (3.1%)	1 (1.1%)
BARC type 1	1 (0.8%)	0	1 (1.1%)
BARC type 2	11 (8.7%)	2 (6.3%)	9 (9.6%)
BARC type 3a	3 (2.4%)	1 (3.1%)	2 (2.1%)
BARC type 3b	0	0	0
BARC type 3c	0	0	0
BARC type 5	0	0	0

## Discussion

The present analysis of the prospective POMPEII study was designed to assess PD profiles in patients undergoing PCI and receiving cangrelor stratified by clinical presentation with or without STEMI. The main findings are as follows:

- Cangrelor-induced platelet inhibition was lower, while rates of HRPR were higher in patients with STEMI compared with those without STEMI.
- After cangrelor discontinuation, platelet inhibition was greater, and rates of HRPR were lower in patients with STEMI compared with those without STEMI. All STEMI patients were switched to ticagrelor, while most patients without STEMI were switched to clopidogrel, indicating that transitioning from cangrelor to ticagrelor might mitigate the risk of platelet activation rebound.
- Among STEMI patients, compared with ticagrelor, cangrelor-induced platelet inhibition was lower with slightly higher rates of HRPR.

To the best of our knowledge, this is the first study demonstrating that cangrelor is less potent among STEMI patients and less potent than ticagrelor.

From a pathophysiological standpoint, it is well-known that STEMI arises from acute thrombotic coronary occlusion, characteristically prompted by atherosclerotic plaque rupture or erosion.^5,6^ This process results in vascular endothelial changes, producing a cascade of platelet adhesion, activation, and aggregation, ultimately leading to thrombosis, culminating in sudden and persistent arterial occlusion. Thrombi are rich in platelets and are stabilized by fibrin. The thrombotic burden in STEMI is huge, determining coronary artery occlusion, which, in turn, leads to myocardial injury that spreads from the subendocardial to the subepicardial myocardium, resulting in a transmural infarction that produces ST-segment elevation on surface ECG. Conversely, NSTEMI is characterized by thrombosis with partial occlusion. Yet, while STEMI is almost always triggered by this mechanism, NSTEMI can result from a wider range of aetiologies, leading to a more heterogeneous pathophysiology. Therefore, inhibition of platelet aggregation has a greater clinical relevance in patients with STEMI compared with NSTEMI and even more compared with unstable angina and CCS undergoing PCI. ST elevation myocardial infarction patients may show reduced platelet inhibition due to various mechanisms (i.e. increased platelet turnover, elevated ADP release, higher circulating catecholamines, microvascular thrombus burden). ST elevation myocardial infarction patients with suboptimal platelet inhibition during primary PCI have worse procedural success and outcomes.^16^ Despite this pathophysiological background, there is not much difference in the clinical practice and literature evidence regarding the type of drug and eventually different doses according to clinical presentation.^1,17^ All patients undergoing PCI should receive aspirin plus a P2Y12 inhibitor (ticagrelor or prasugrel recommended over clopidogrel in ACS only). If patients are P2Y12 inhibitor naïve, they may receive cangrelor before PCI to reach a more rapid antiplatelet effect, which in ACS, especially in STEMI, is of paramount relevance considering the above-mentioned role of platelets and thrombosis. Hence, adequate inhibition of platelet aggregation is fundamental, yet it is unclear whether differences in terms of magnitude of platelet inhibition exist among different clinical presentations in patients treated with same dose of cangrelor during PCI.

The facilitation through aggrastat or cangrelor bolus and infusion over prasugrel: a multicentre randomized open-label trial in patients with ST-elevation myocardial infarction referred for primary percutaneous intervention (FABOLUS-FASTER) trial has generated debate in terms of cangrelor-induced amount of platelet inhibition and relevant rates of on-treatment HRPR as assessed with the LTA.^18–22^ ST elevation myocardial infarction patients undergoing primary PCI were enrolled, and IPA induced by cangrelor, tirofiban, and prasugrel was compared.^10^ Based on preclinical PD data that had shown very high degrees (>80%) of platelet inhibition induced by cangrelor,^23^ the primary hypothesis of the trial was that cangrelor could be non-inferior to tirofiban. However, the trial did not confirm this hypothesis, while even showing that cangrelor was inferior to tirofiban (IPA of 34.1% vs. 95.0%) and was associated with around 50% of HRPR as assessed by LTA stimulated with ADP 20 μM.^15^ Additionally, other PD studies using different platelet function assays showed contrasting data.^2,7–9,14^ Therefore, POMPEII study was designed to obtain contemporary data with different assays of platelet aggregation in all clinical settings.^14^ Overall, POMPEII study showed a greater IPA than that observed in FABOLUS-FASTER, with limited, but present, HRPR during cangrelor infusion at LTA, but IPA was moderate and well below 80% in most patients.^14^ However, such apparent discrepancies should be interpreted in light of the differences among patients enrolled. Indeed, here we explored whether some effects could be related to the presentation with STEMI showing that these patients had a significantly lower cangrelor-induced effect compared with patients without STEMI.

The use of cangrelor includes the need to switch to an oral P2Y12 inhibitor during or after its interruption. Given their different pharmacological properties, drug–drug interactions could occur during concomitant treatment and the optimal transition strategy has been widely investigated.^11–13^ While prasugrel and clopidogrel should be administered at the end of the cangrelor infusion to prevent drug–drug interactions, ticagrelor can be administered concomitantly given its longer half-life making it available for receptor binding after cangrelor interruption. In the POMPEII study, we have previously shown that the transition from cangrelor to oral drugs could expose many patients to a variable period of on-treatment HRPR and that this happened predominantly with clopidogrel but not with ticagrelor.^4,14^ Here, we observed that patients with STEMI, who all received ticagrelor during cangrelor infusion, had adequate platelet inhibition with limited HRPR. On the contrary, patients without STEMI, who predominantly switched to clopidogrel at the end of infusion, had high rates of HRPR. Recently, ticagrelor has been suggested to be the preferential drug for long-term monotherapy.^24^ Our data further corroborate the preferential use of ticagrelor even in the periprocedural phase when cangrelor is used, and its coadministration as soon as possible to accelerate its effects while limiting delays and potential platelet aggregation rebound. In the setting of CCS undergoing elective PCI, most recent ESC guidelines do not mention cangrelor anymore, and clopidogrel continues to be the most used oral P2Y12 inhibitor.^17^ However, some operators may consider cangrelor use in complex PCI,^2,4^ but they should be aware of these data showing risks of switching to clopidogrel.

Interestingly, among STEMI patients we compared the IPA at 30 min during cangrelor with that at 4–6 h when IPA was totally related to ticagrelor effect. Although ticagrelor might have not yet reached its maximum effect given the well-known delayed effects of oral P2Y12 inhibitors during ACS, the IPA induced by ticagrelor was significantly higher than cangrelor. This finding is in line with that observed in the FABOLUS-FASTER trial, in which the IPA during cangrelor was significantly lower compared with that induced by prasugrel.^10^

Pharmacodynamic data may inform and guide clinical practice and future research. Our data suggest that attention should be paid when cangrelor is used in all clinical settings, particularly in STEMI, considering that it could be associated with poor responsiveness, and with eventual platelet aggregation rebound, particularly when transitioning is not done with ticagrelor. Future studies should further investigate these issues and alternative strategies.

### Limitations

Although the lower platelet inhibition in STEMI patients may raise concerns, the study is underpowered for clinical events and cannot assess clinical implications of the PD findings.

This is a single-centre study, but this also represents a strength by reducing variability of PD assessments.

The use of cangrelor and the selection of type and timing of oral P2Y12 inhibitor were left at operators’ discretion introducing some bias; however, this also mirrors the daily practice and new scientific evidence (i.e. the early administration of ticagrelor after cangrelor despite not yet approved in Europe based on the encouraging data from the CANTIC trial).

Finally, some selection bias is related to enrolment of nonconsecutive patients; however, this reflects the fact that cangrelor is not routinely used in all consecutive patients and that PD analysis requires a dedicated team available for up to 6 h.

Therefore, our explorative findings should be interpreted with caution and considered hypothesis-generating, thus requiring further investigations.

## Conclusions

In a contemporary treated population of patients with or without STEMI undergoing PCI, this study provides PD data showing that cangrelor-induced platelet inhibition was lower in patients with STEMI compared with those without STEMI and was lower compared with that induced by ticagrelor among STEMI patients. The switch from cangrelor to an oral P2Y12 inhibitor exposed patients without STEMI to greater HRPR compared with those with STEMI, supporting the preferential use of ticagrelor for switching when cangrelor is used to mitigate the risk of platelet aggregation rebound.

## Supplementary Material

zuag056_Supplementary_Data

## Data sharing

Data sharing will be available upon reasonable request to the corresponding author.
