# Supplementary material for: Pharmacodynamic effects of cangrelor in patients with or without STEMI undergoing percutaneous coronary intervention: insights from the POMPEII study
Source: Eur Heart J Acute Cardiovasc Care. 2026 Apr 15;15(7):524–34. doi: 10.1093/ehjacc/zuag056 (PMC13344179; doi:10.1093/ehjacc/zuag056)
Supplement: zuag056_Supplementary_Data [file zuag056_supplementary_data.docx]

**Pharmacodynamic effects of cangrelor in patients with or without STEMI undergoing percutaneous coronary intervention: insights from the POMPEII study.**

Giuseppe Gargiulo, Viviana Narciso, Imma Forzano, Luca Sperandeo, Domenico Simone Castiello, Fiorenzo Simonetti, Lina Manzi, Domenico Florimonte, Mario Enrico Canonico, Marisa Avvedimento, Roberta Paolillo, Alessandra Spinelli, Luigi Di Serafino, Carmen Anna Maria Spaccarotella, Anna Franzone, Raffaele Piccolo, Eugenio Stabile, Plinio Cirillo, Marco Valgimigli, Giovanni Esposito.

**Supplementary Appendix**

**SUPPLEMENTARY RESULTS**

***PD assessments with medians with interquartile range (IQR) and compared through Mann-Whitney U test.***

Results were entirely consistent when analyses were conducted by medians with IQR and compared with a non-parametric test (Mann-Whitney U test).

At 30-minute, during cangrelor infusion, the median IPA at LTA 20-μM-ADP was 52.0 (44.1–59.8) versus 60.9 (50.2–71.6) in patients with and without STEMI, respectively (p=0.013). At LTA 5-μM-ADP was 58.3 (51.5–65.8) versus 65.6 (58.1–72.4) in patients with and without STEMI, respectively (p=0.011). MEA-AUC was 25.0 (19.0–30.5) versus 21.0 (17.2–29.0), respectively (p=0.17). PRU was 38.5 (26.8–67.0) versus 20.5 (8.0–50.0), respectively (p=0.01).

At 3h, the median IPA at LTA 20-μM-ADP was 58.8 (42.6–67.6) versus 36.1 (14.7–71.4) in patients with and without STEMI, respectively (p=0.057). At LTA 5-μM-ADP was 63.6 (45.3–78.3) versus 41.4 (17.5–75.3) in patients with and without STEMI, respectively (p=0.022). MEA-AUC was 22.0 (12.5–38.0) versus 28.0 (17.0–48.0), respectively (p=0.027). PRU was 43.0 (5.5–74.5) versus 139.0 (10.0–209.0), respectively (p=0.002).

At 4-6h, the median IPA at LTA 20-μM-ADP was 66.3 (45.1–75.7) versus 59.6 (30.1–78.0) in patients with and without STEMI, respectively (p=0.29). At LTA 5-μM-ADP was 70.0 (58.0–84.8) versus 67.9 (37.5–87.2) in patients with and without STEMI, respectively (p=0.49). MEA-AUC was 15.0 (10.0–25.0) versus 19.0 (13.0–31.0), respectively (p=0.127). PRU was 20.0 (6.0–45.0) versus 72.0 (5.0–156.0), respectively (p=0.027).

***PD assessment of LTA with linear mixed-effects model to assess the temporal evolution of IPA.***

The linear mixed-effects model analyses confirmed that the rebound effect was significant in patients without STEMI, but not in those with STEMI.

Both with LTA 20-µM and 5-µM ADP stimulation, we observed a significant reduction of IPA from 30 minutes to 3h and to 4-6h in patients without STEMI while minimal variation in patients with STEMI, resulting in significant time × presentation interactions at both 3h (20-µM ADP: p<0.001; 5-µM ADP: p<0.001) and 4-6h (20-µM ADP: p=0.003; 5-µM ADP: p=0.02). Overlapping results were observed with MEA-AUC (3h: p=0.0002; 4-6h: p=0.001) and PRU(3h: p<0.0001; 4-6h: p<0.0001).

***PD assessment in STEMI compared with Chronic coronary syndrome (CCS).***

At 30 minutes, during cangrelor infusion, the IPA at LTA 20-μM was 51.5±16.2% in STEMI vs 58.8±17.9% in CCS, p=0.049; at LTA 5-μM was 58.1±14.3% in STEMI vs 63.5±15.6% in CCS, p=0.09; at MEA AUC was 26.7±12.7 in STEMI vs 21.9±9.1 in CCS, p=0.039; at VerifyNow PRU was 55.4±51.5 in STEMI vs 28.8±32.9 in CCS, p=0.01.

At 3h and 4-6h, IPA was lower and AUC and PRU were higher in CCS patients indicating a greater rebound effect compared with STEMI (all p values <0.01).

Also HRPR rates were overall consistent being greater in STEMI at 30 minutes with all tests (LTA 20-μM: 6.3% vs 3.1%, p=0.4; LTA 5-μM: 6.3% vs 1.6%, p=0.2; AUC and PRU: 6.3% vs 0%, p=0.04) and lower at 3h (LTA 20-μM: 19.4% vs 60.3%, p<0.0001; LTA 5-μM: 16.1% vs 58.7%, p<0.0001; AUC: 3.2% vs 47.6%, p<0.0001; PRU: 6.5% vs 3.3%, p=0.004) and 4-6h (LTA 20-μM: 3.2% vs 25.4%, p=0.009; LTA 5-μM: 6.5% vs 25.4%, p=0.02; AUC: 3.2% vs 47.6%, p<0.0001; PRU: 0% vs 7.9%, p=0.1) compared with CCS group.
